# Evaluation of the Wound Healing Potential of *Cynara humilis* Extracts in the Treatment of Skin Burns

**DOI:** 10.1155/2023/5855948

**Published:** 2023-04-18

**Authors:** Najoua Salhi, Otman El Guourrami, Lamiae Rouas, Siham Moussaid, Amina Moutawalli, Fatima Zahra Benkhouili, Mouna Ameggouz, Mohammed Merae Alshahrani, Ahmed Abdullah Al Awadh, Abdelhakim Bouyahya, My El Abbes Faouzi, Yahya Cherrah

**Affiliations:** ^1^Pharmacoepidemiology and Pharmacoeconomics Research Team, Laboratory of Pharmacology and Toxicology, Faculty of Medicine and Pharmacy, Mohammed V University in Rabat, Rabat, Morocco; ^2^Laboratory of Analytical Chemistry and Bromatology, Faculty of Medicine and Pharmacy, Mohammed V University in Rabat, Rabat, Morocco; ^3^Laboratory of Anatomy Cytology, Faculty of Medicine and Pharmacy, Children's Hospital, Mohammed V University in Rabat, Rabat, Morocco; ^4^Laboratory of Plant, Animal and Agro Industry Productions, Faculty of Science, Ibn Tofail University, B.P 133, Kenitra 1400, Morocco; ^5^Department of Drug Sciences, Laboratory of Medicinal Chemistry, Faculty of Medicine and Pharmacy, Mohammed V University in Rabat, Rabat, Morocco; ^6^Department of Clinical Laboratory Sciences, Faculty of Applied Medical Sciences, Najran University, Najran 61441, Saudi Arabia; ^7^Laboratory of Human Pathologies Biology, Faculty of Sciences, Department of Biology, Mohammed V University in Rabat, Rabat, Morocco; ^8^Biopharmaceutical and Toxicological Analysis Research Team, Laboratory of Pharmacology and Toxicology, Faculty of Medicine and Pharmacy, Mohammed V University in Rabat, Rabat, Morocco

## Abstract

*Cynara humilis* is traditionally used to treat skin burns and microbial infections. However, experimental studies on this plant are rare. Furthermore, the aim of this study was to investigate the effects of *Cynara humilis*, a Moroccan herbal remedy, on the healing of deep second-degree burns in rats with a silver sulfadiazine group. This research was also carried out to confirm if *C. humilis* had antibacterial capabilities. Under typical burn procedures, each rat received a deep second-degree burn on the upper back. The burns were treated regularly with control groups (control and control VH), silver sulfadiazine (SDD) in group 3, *C. humilis* ethanolic extract (CHEE) in group 4, and *C. humilis* aqueous extract (CHAE) in group 5. Throughout the treatment, digital photography was used to measure rat responses to the treatment until day 18. After the scar biopsy at the end of the study, histological parameters (inflammatory cells, collagen, epithelialization, fibrosis, and granulation tissue) were assessed. Using the well technique, the antibacterial activity of the extracts was tested against *Staphylococcus aureus* CIP 483, *Bacillus subtilis* CIP 5262, *Escherichia coli* CIP 53126, *Pseudomonas aeruginosa* CIP 82118, and *Salmonella enterica* CIP 8039, and the results showed important activities of the ethanolic and aqueous extracts against the five species tested with MICs of 2 and 4 mg/mL, respectively. In the aqueous extract group, the wound healed faster. In addition, the healing rate in the *C. humilis* extracts (CHEA and CHEE) group was faster than in the silver sulfadiazine and control groups. In the *C. humilis* group, maximum wound surface recovery was observed at the same time, as it was not noted in the silver sulfadiazine group. Pathologically, epithelialization was more marked in wounds treated with *C. humilis* extracts (CHE). Angiogenesis and inflammatory cells were considerably lower in the CHE group than in the silver and other control groups. However, elastic fibers were considerable in the CHE-treated group. In histological examination, the *C. humilis* group had a low incidence of angiogenesis and inflammation, indicating that this group had less wound scarring. Collagen and burn wound healing were both faster in the *C. humilis* group. The findings of this study suggest that *C. humilis*, as indicated by traditional medicine, is a promising natural source for the management of wound healing.

## 1. Introduction

Burns are a global public health problem. Indeed, every year, millions of people suffer from skin burns all over the world [1]. Given the importance of skin as a barrier structure protecting the organism from external shocks, particularly infectious ones, postburn sequelae are one of the most problematic disorders in the medical world [2].

Over 90% of fatal burns from open fires occur in developing countries, with the poorest and middle-income populations bearing the brunt of the toll [3]. Burn-related mortality is estimated at approximately 265,000 deaths per year worldwide [4]. The medicinal plants and native trees have been used in burn treatments and other natural remedies such as astringent properties and antibacterial effects [5].

The creation of medicines that efficiently and quickly heal damaged tissues while reducing treatment expenses greatly improves patients' quality of life. Phytotherapy, which uses the extract of different parts of plants, is one of the therapies employed. Nature has given medicinal agents, and an incredible number of contemporary medications have been identified from natural sources, based on their use in traditional medicine [6].

Certainly, the wound healing effect of medicinal plants is due to their diverse bioactive components such as terpenoids, flavonoids, tannins, and phenols. Herbal medicines had other advantages, including easy accessibility, fewer side effects, and low cost [7].

Decades ago, humans have used various portions of plants in the treatment and prevention of numerous illnesses since the dawn of time [8–11]. Opium, *Physostigma venenosum* (Eserine), cinchona bark, and cocaine are examples of herbal medicines that have been historically important for centuries [12].

Therapeutic medicine arose as a result of scientific advances, and the notion of relying on traditional treatment began to fade. However, in many rural areas, the lack of hospitals or healthcare units, the high cost of pharmaceuticals, and the insufficiency of modern treatments to effectively cure some human diseases have forced the rural population to rely on traditional and herbal medicines for their healthcare [5, 13].

The hunt for the antibacterial activity of natural extracts has intensified. Natural biocides derived from higher and aromatic plants have long been used to control bacterial and fungal pathogens that cause disease in both humans and plants [14].

Antibiotics have revolutionized human health by allowing the treatment of life-threatening infections [15]. However, with the rise in bacterial resistance to currently available antibiotics, it is now more important than ever to hunt for innovative medicines derived from natural sources [16].

Medicinal herbs are widely used in Morocco, despite the lack of data in the literature. Indeed, the few local ethnopharmacological studies on medicinal plants used to treat skin burns showed 36 medicinal plants that are utilized to cure skin burns in various Moroccan localities [17–19].

The current study focused on the Moroccan medicinal plant, namely *Cynara humilis* belonging to the Asteraceae family. *C. humilis* is very known by wild thistle, native to the Mediterranean region and traditionally used as a coagulant in the artisanal production of sheep's and goat's cheeses, and against burns for the healing process [17–20]. Ethnomedicinal surveys of *C. humilis* have indicated that the powder is most used by locals in Morocco. It is used as an anti-injury material [19], astringent, wound healing, and anti-infective agents.

However, to the best of our knowledge, the antibacterial activities of *C. humilis* roots have been examined in only one recent study [21]. The aim of this study was to determine the wound healing and antibacterial effects and to evaluate the treatment of skin burns with *C. humilis* extracts.

## 2. Material and Methods

### 2.1. Plant Collection and Extraction

**Figure 1:**
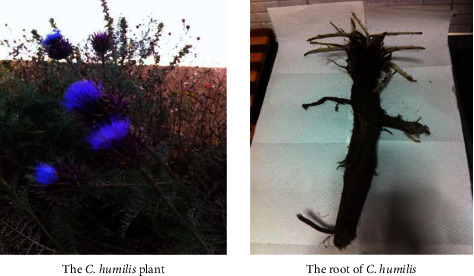
Root of *Cynara humilis*.

The *C. humilis* roots (Figure 1) were collected from the Skhirat region (Morocco) on April 2018. The medicinal plant *C. humilis* was selected based on ethnopharmacological information from traditional herbalists in the region. The identification of *C. humilis* was carried out, and the voucher specimen was deposited in the herbarium of the Scientific Institute of Rabat in Morocco according to the code (RAB79161).

The powder (250 g) of *C. humilis* root was macerated in one liter of distilled water under magnetic stirring for 24 h, at room temperature, and protected from light. The extracts were filtered on Whatman paper and then concentrated under reduced pressure in the rotavapor at 50°C. The residue obtained was freeze-dried to remove all traces of solvent, and stored at +4°C. The total aqueous extract (CHAE) and the ethanolic extract (CHEE) were prepared in the same way.

### 2.2. A Percutaneous Salve Preparation

The plant extracts in this study were used as a 20% ointment. They were combined with inactive ingredients that function as a moisture-retaining patch, enhancing skin hydration and facilitating the absorption of active components. The extracts were dissolved in purified water and thoroughly mixed. Afterwards, in a mortar preheated in a water bath at 70°C and with the help of a pestle, lanolin was triturated with each prepared extract solution and added in small fractions until their total absorption. Then, Vaseline was carefully incorporated into each mixture until a homogeneous ointment was obtained (Table 1). The prepared ointments were stored in sterile bottles in the refrigerator at +4°C.

### 2.3. Experimental Animals

For our study, a total of 30 Wistar rats composed of male and female adults, weighing between 180 g and 300 g were used for dermal toxicity. A total of 30 adult male Wistar rats, weighing between 180 g and 250 g, were utilized for the wound healing investigation. The female rats were nulliparous and not pregnant, and all the animals had healthy and intact skin.

The animals used in this study were housed in cages and acclimated at a temperature of 22°C (±3), at a relative humidity between 30% and 70%, and subjected to a light/dark cycle of 12/12 hours.

### 2.4. Antimicrobial Activity

Antimicrobial activities of the plant extracts were determined using five standard bacterial strains from the Institut Pasteur Collection (CIP): *Staphylococcus aureus* CIP 483, *Bacillus subtilis* CIP 5262, *Escherichia coli* CIP 53126, *Pseudomonas aeruginosa* CIP 82118, and *Salmonella enterica* CIP 8039.

The revival of the strains was done by adding to 100 *μ*L strains preserved in glycerol at +4°C, 10 mL of tryptone soy broth TSB (tryptic soy broth), and incubation of the media for 18 hours at 37°C. For the minimum inhibitory concentration (MIC), a modified resazurin microtitre-plate assay was used as reported by Sarker et al. [22] and Marmouzi et al. [23].

Under aseptic conditions, serial dilution was achieved by transferring 100 *μ*L of the test material from the first row to successive wells of the next row of the same column, then dosing 100 *μ*L of the material at sequential drop concentrations into each well.

After adding 10 *μ*L of a bacterial suspension (10^8^ CFU/mL) to each well, the microplates were incubated at 37°C for 24 hours. A column containing an antibiotic was used as a positive control (chloramphenicol in sequential dilution at a concentration of 25 mg/mL). For all plant extracts in suspension, the concentrations used were 32 mg/mL.

Finally, a resazurin solution was prepared by dissolving 270 mg of resazurin powder in 40 mL of sterile distilled water. The solution was stirred until the powder was completely dissolved and the solution became homogeneous [24]. After incubation, a volume of 10 *μ*L of resazurin solution (5 *μ*m/mL) was added to each well. Then, the microplates were placed in a 37°C incubator for only 2 hours. The color shift was then visually examined. Any color changes from purple to pink or colorlessness were considered positive. The MIC values were determined by taking the lowest concentrations at which color change occurred.

### 2.5. Dermal Toxicity

Acute dermal toxicity of *C. humilis* extracts was performed on 30 Wistar rats comprising males and females according to the OECD 402 limit test (OCDE, 1987) at a dose of 5000 mg/kg body weight. 24 hours before the test, using an electric tensioner, the dorsal region of the trunk of the animals was shaved, in which this area presented 10% or more of the total body surface in order to avoid skin lesions. The animals were divided into three groups of 10 rats each, with an equal number of males and females (*n* = 10).

The control group was treated with the vehicle (water), and the test groups were treated with the aqueous and ethanolic extracts. The previously weighed dose was moistened with water and then applied to the shaved part of the animal by performing a slow massage to increase the penetration of the product. The area was then covered with gauze and held in place with a bandage for 24 hours. Then, the dressing and the gauze were removed, and the skin was cleaned with lukewarm water and then each animal was returned to its individual cage.

After substance application, the rats were observed every 30 minutes for 10 hours on the first day and daily for 14 days. During this observation period, the weight of each animal was measured once a week, and the behavioral manifestations, the signs of local and systemic toxic symptoms, and the number of deaths were recorded daily.

### 2.6. Wound Healing Properties

The rats were anesthetized by injecting 100 mg/kg ketamine hydrochloride intraperitoneally, and their backs were shaved using an electric clipper and disinfected with 70% ethanol. A deep-thickness second-degree skin burn on the back of the rats corresponding to a 3.14 cm^2^ was performed based on earlier studies with minor modifications [25].

The rats were randomly divided into five groups of six animals in each group as follows:

  Control group: animals induced with a wound not treated with any drug
  VH control group: animals treated with the excipients of the containing ointment (purified water, petroleum jelly, and lanolin)
  SSD Group: animals treated with the standard silver sulfadiazine 1% ointment
  CHEE group: animals treated with an ointment containing 20% of the ethanolic extract of *C. humilis*
  CHEE group: animals treated with an ointment containing 20% of the aqueous extract of *C. humilis*

Wound treatment was performed immediately after induction of the burn and once a day until the 18th day. Approximately, 0.5 g of each treatment was evenly applied to the wound to cover the entire burned area. The burned animals have been caged individually.

#### 2.6.1. Retraction Evolution Protocol

The clinical evaluation of the animals has been conducted by monitoring their weight every three days. The clinical evolution of the wounds was carried out daily in the morning by macroscopic examination of the lesions throughout the healing period of the burns. This examination is based on the observation of the appearance of changes in the color, odor, and appearance of the wounds, the formation of edema, bubbles, or crusts, bleeding, swelling, and secretion.

Wound healing was assessed by calculating the rate of wound retraction. For this purpose, the wound surfaces were measured at *D*_6_, *D*_12_, and *D*_18_ days after burn induction.

The burn site was photographed using a Canon SX710 HS digital camera (20.3 megapixels; zoom = 1). In parallel, a direct manual tracing of the wound surfaces on transparencies was carried out. The photos and transparencies were analyzed by image processing software (ImageJ software) to calculate the surfaces and determine the retraction rate. The wound retraction rate was calculated by the following formula according to Senthil Kumar et al. [5].

(1) $$\begin{array}{c} Percent\;wound\;retraction\;of\;J=\frac{Initial\;wound\;size\;J0-specific\;day\;wound\;size\;Jx}{Initial\;wound\;size\;J0}\times 100\text{.} \end{array}$$

#### 2.6.2. Histological Analysis

In this study, light microscopy was used to examine the histology of the skin. The histological evaluation was carried out at the end of the experiment on day 18 after the sacrifice of all the animals under ethical conditions. The biopsies were performed by taking a sample from the scar using a scalpel. Fixation was performed with 10% neutral buffered formalin (NBF) for 24 h at 4°C. After being dehydrated in alcohol, the skin samples were embedded in paraffin wax (58–60 mp). 4 *µ*m thick skin sections were stained with hematoxylin, green trichrome from Masson, and orcein. Stained skin slices were shot with a camera linked to a photo microscope.

The degree of inflammatory response, collagen and elastic fiber deposition, angiogenesis, granulation tissue development, and epithelialization were all measured histologically to determine wound healing capacity. Each parameter was given a score ranging from 0 to 3 [26]. Masson's trichrome green staining and orcein staining were used for qualitative estimation of collagen deposition and elastic fibers, respectively.

### 2.7. Statistical Analysis

The statistical analysis was performed using RStudio 4.6 software, and data were expressed as the mean ± standard deviation (SD) for each measurement. The data were also analyzed by one-way analysis of variance (one-way ANOVA). Posthoc procedure was used for the significance of difference (*p* < 0.05).

## 3. Results

### 3.1. Antibacterial Activity

In our study, the antibacterial activity of two extracts from the roots of *C. humilis* was studied against five bacterial species. The results (Table 2) showed that the ethanolic extract possesses antibacterial activities against *S. aureus* CIP 483, *E. coli* CIP 53126, *B. subtilis* CIP 5262, *S. enterica* CIP 8039, and *P. aeroginosa* CIP 82118 at a similar minimum inhibitory concentration (MIC) equal to 2 mg/mL for all bacterial species. The aqueous extract also revealed antibacterial activity against the five bacterial species at MICs equal to 4 mg/mL but lower than those of the ethanolic extract.

### 3.2. Acute Dermal Toxicity

**Figure 2:**
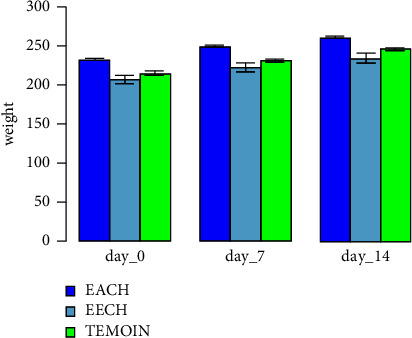
Evolution of body weight of animals in acute dermal toxicity of *C. humilis* extracts treatment.

From day 0 to day 14, the body weights of both the control and treatment groups were gradually increased, as shown in Figure 2. There was no significant difference in average weight between the two groups. During the 48-hour observation period, no meaningful clinical symptoms of *C. humulis* toxicity at a dosage of 5 g/kg were noticed. On the other hand, the animals stayed normal for the next 14 days and no death was observed.

### 3.3. Body Weight Evolution (in Burn Study)

**Figure 3:**
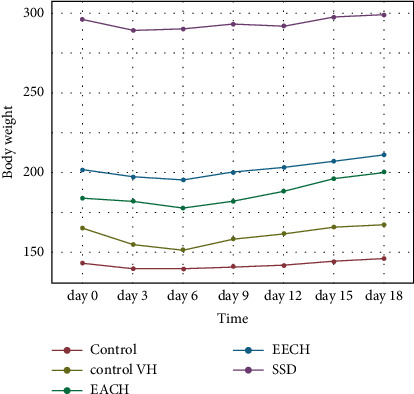
Evolution of body weight of animals treated with *Cynara humilis* extract, SSD, control VH, and control in burn study. VH: vehicle; SSD: silver sulfadiazine.

Figure 3 represented the evolution of the average body weights of the rats receiving the different treatments every three days. The mean body weight of rats in the control VH and SSD groups was not statistically different (*p* < 0.05) from the control group. The gain in body weight during the experimental period (*D*_0_ to *D*_18_) was 3 g, 2.33 g, and 2.67 g, respectively, for control, control VH, and SSD.

Unlike to the two groups treated with plant extracts, the weight of the rats having received CHAE was increased from 184.17 g on day 0 to 200.5 g on day 18, representing a gain in body weight of 16.33 g. From 202.0 g (day 0) to 211.17 g (day 18) for the CHEE group rats, a body weight gain of 9.17 g was obtained.

### 3.4. Macroscopic Evaluation of Rat Wounds by *Cynara humilis*

**Figure 4:**
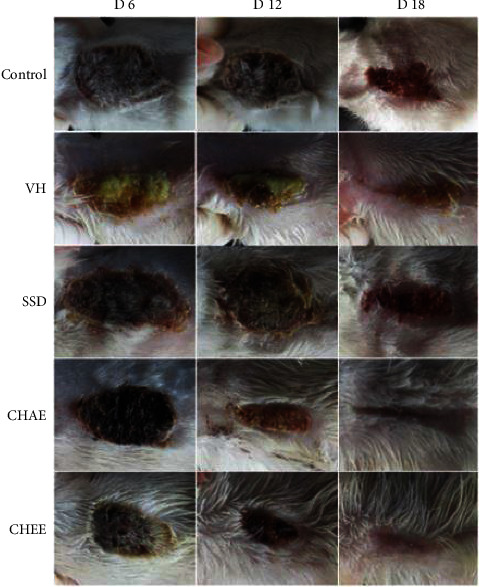
Macroscopic observation in burn wounds after 18 days in not treated and treated rats (CHAE, CHEE, SSD, and vehicle control). VH: control vehicle; SSD: silver sulfadiazine.

Topical use of *C. humilis* compounded into an ointment base on wound healing in rats resulted in a considerably (*p* < 0.05) greater rate of wound healing in rats in the current investigation. The wound area after *C. humulis* ointment administration was indicated in Figure 4. Wistar rats treated within 12–18 days had a superior healing pattern with full wound closure (Figure 4), whereas the control group needed more than 18 days.

### 3.5. Contraction Evolution Results during 18 Days of Treatment

**Figure 5:**
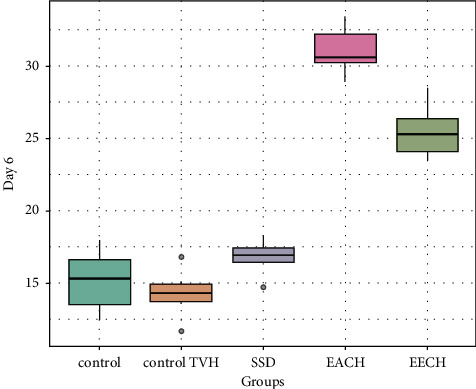
Retraction rate in treated groups by *C. humulis* in 6 days. TVH: control vehicle; SSD: silver sulfadiazine.

**Figure 6:**
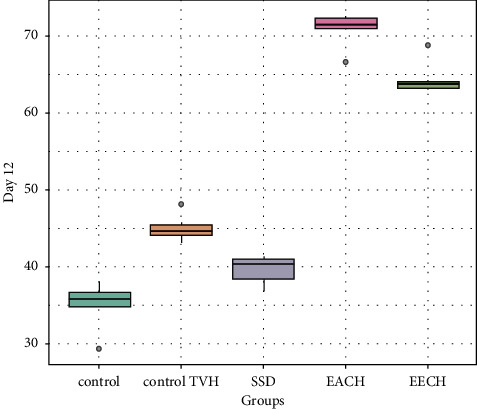
Retraction rate in treated groups by *C. humulis* in 12 days. TVH: control vehicle; SSD: silver sulfadiazine.

**Figure 7:**
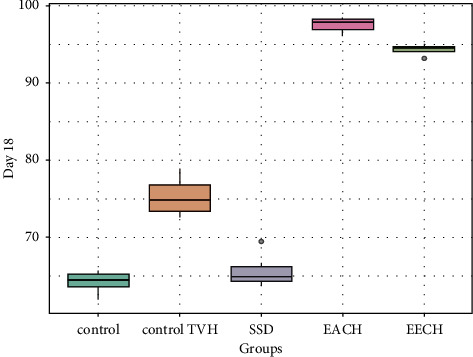
Retraction rate in treated groups by *C. humulis* in 18 days. TVH: control vehicle; SSD: silver sulfadiazine.

Figures 5–7 demonstrated the differences in wound area assessed on days 6, 12, and 18, after burn in the three treatment groups compared to the two control groups. Eighteen days after the burn, the extract EECH- and EACH-treated groups had achieved more than 90% wound healing. The CHAE-treated group had achieved a healing rate of 32% on day 6 and 71% wound healing after 12 days, which was not substantially different (*p* > 0.001) from the group treated with the CHEE extract. The percentage of healing in the SSD-treated group was substantially lower (*p* < 0.001) than in the extract-treated group, with 65.7% on day 18. It can be seen that the wound contraction percentage of *C. humilis* ethanolic extracts (97.58%) and aqueous extract (94.31%) after 18 days is substantially higher (*p* < 0.001) compared to untreated group (64.2%) and the groups treated with vehicle control (75.3%).

### 3.6. Histological Results

**Figure 8:**
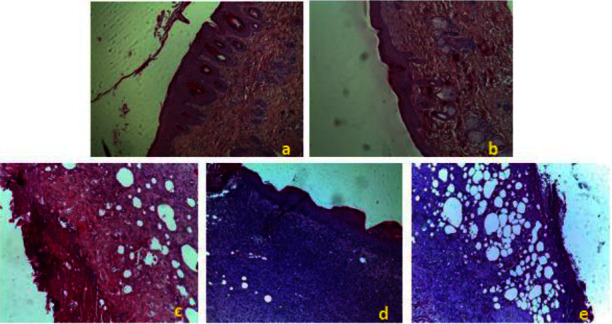
Histopathological change examination of coloration with hematoxylin and eosin of the skin of control and treated groups of rats. (a) EACH; (b) EEAH; (c) Control; (d) TVH control; (e) SSD. TVH: control vehicle; SSD: silver sulfadiazine.

**Figure 9:**
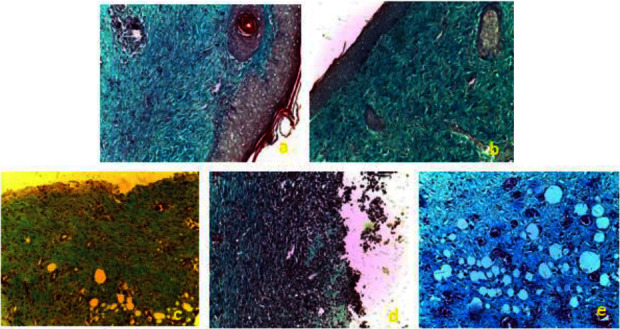
Histopathological change examination of coloration with green trichrome from Masson of the skin of control and treated groups of rats. (a) EACH; (b) EEAH; (c) Control; (d) TVH control; (e) SSD. TVH: control vehicle; SSD: silver sulfadiazine.

**Figure 10:**
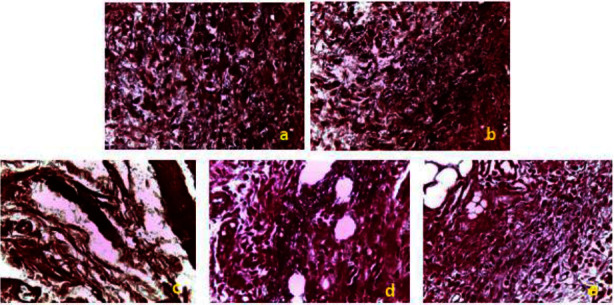
Histopathological change examination of coloration with orcein of skin of control and treated groups of rats. (a) EACH; (b) EEAH; (c) Control; (d) TVH control; (e) SSD. TVH: control vehicle; SSD; silver sulfadiazine.

On the 18th day of treatment after induction of the burns, the histological evaluation of the wounds was carried out using three types of staining. Figures 8–10 shows histological sections of wound scars observed under an optical microscope. The control, control VH, and SSD groups showed nonreepithelialized epidermis after 18 days of treatment. The dermis showed ulceration with the presence of necrotic tissue debris mixed with polymorphic inflammatory cells with congestion and edema (Figure 8).

The treatment of rats with *C. humilis* extracts resulted in significant epithelialization and a decrease in inflammatory cells with the absence of necrotic tissue debris. There was also the production of a modest amount of extracellular matrix and the development of new blood vessels and hair follicles (Figure 8).

The study of collagen fiber deposition and organization was conducted using Masson's Trichrome Vert staining for histological evaluation (Figure 9). The results revealed low density and poor organization of collagen fibers in rats from the control, control VH, and SSD groups, along with persistent epidermal ulceration and a polymorphic inflammatory infiltrate in the dermis with edema, indicating an incomplete healing process (Figure 9).

The groups treated with *C. humilis* extracts showed an improvement in wound healing, as evidenced by a high density and better organization of collagen, as revealed by the analysis of collagen quantity and quality (Figure 9).

The deposition of elastic fibers was analyzed after staining the histological sections with orcein. Under an optical microscope, the elastic fibers were visible as wavy bundles, sometimes connected, with a reddish-brown color on a beige background (Figure 10). The results showed the abundance of elastic fibers in the histological sections in the groups treated with *C. humilis* extracts, unlike the other groups.

**Figure 11:**
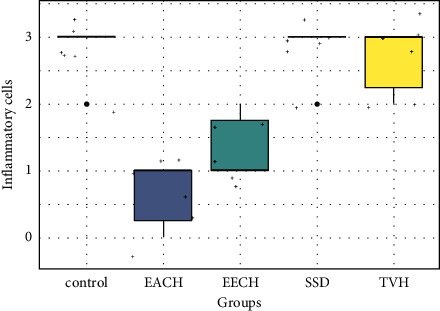
Histopathological scores for inflammatory cells. TVH: control vehicle; SSD: silver sulfadiazine.

**Figure 12:**
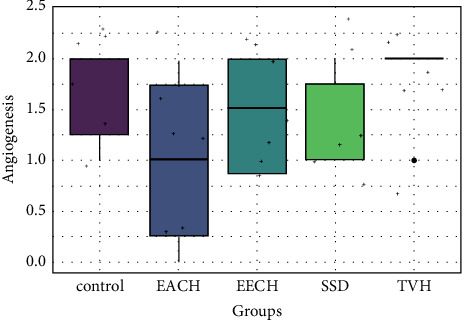
Histopathological scores for angiogenesis.

**Figure 13:**
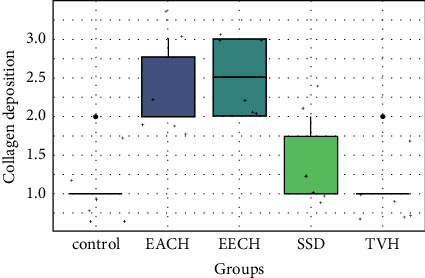
Histopathological scores for collagen.

**Figure 14:**
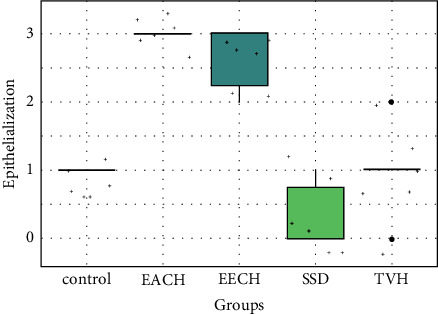
Histopathological scores for epithelialization.

**Figure 15:**
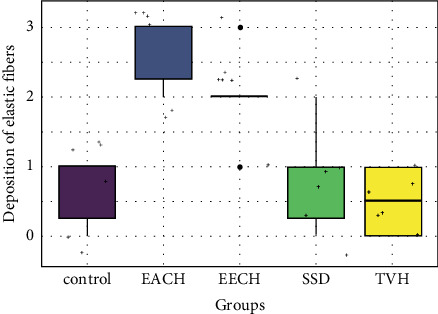
Histopathological scores for elastic fibers.

**Figure 16:**
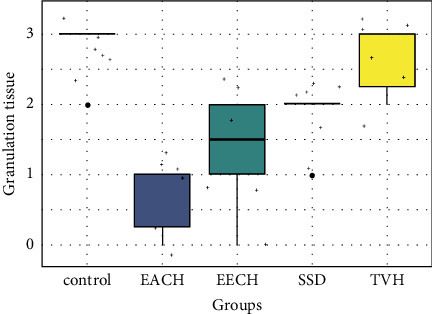
Histopathological scores for granulation tissue.

Histological analysis of wound biopsies was also performed based on a scoring system by studying collagen and elastic fiber deposition, inflammatory response, appearance of granulation tissue, angiogenesis, and epithelialization (Figures 11–16).

In all groups, the histopathological picture is nearly identical. On the eighth day, the number of inflammatory cells was higher in wounds of control, TVH control, and SSD groups than the groups treated with the creams containing 20% of the *C. humilis* extracts (Figure 9).

All groups displayed moderate new blood vessel formation (angiogenesis), with the control and TVH control groups exhibiting modest angiogenesis (Figure 12). The proportion of collagen bundles in microscopic fields was used to score collagen synthesis in the skin. Collagen analysis revealed no significant differences between *C. humilis* extracts (EECH and EACH) and a low score difference between control and TVH control (Figure 13).

However, there is no significant difference in reepithelialization score between the control and TVH control (*p* > 0.05), while there is a significant difference when comparing controls with SDA therapy (*p* > 0.05). On EACH and EECH, the greatest reepithelialization score (Figure 14) was found, and it exhibited comparable significance (*p* < 0.01). The treatment groups (EECH and EACH) had superior reepithelialization observations than the controls (control and TVH) and SSD groups (*p* < 0.05). Collagen quantity and quality were both raised in *C. humilis* extracts. The plant therapy groups had improved inflammation and angiogenesis processes.

The deposition of elastic fibers is highly controlled over the 18 days (Figure 15), and comparable patterns were detected across (control, TVH control, and SDA) with a low significant difference (*p* > 0.05). Low score of elastic fibers was observed in scars that were treated with SDA and control groups. During the 18th day, substantially more elastin fibers were found inside the dermal scar tissue in the two treatment groups that received EECH and EACH in wound healing after burn skin (*p* < 0.05). The volume and distribution of granulation tissue on the group treatments (Figure 16) were considerably lower in the *C. humilis* ointment group (*p* < 0.01) than in the control group (*p* > 0.05). The best wound healing results are shown in EECH and EACH (*C. humilis* treatment), which have a high density of collagen with a good organization, a full and mature epithelium, a low number of inflammatory cells, and angiogenesis, respectively.

## 4. Discussion

Burn wounds are particularly vulnerable to complications [27, 28]. Complications can wreak havoc on the healing process in a variety of ways. An infection is the most likely consequence, according to this study. As a result, the comparison between standard drugs (chloramphenicol) and topical drugs (*C. humilis* extracts ointment) revealed that topical drugs were highly active and similar to chloramphenicol against Gram-negative and Gram-positive bacteria, as well as *Pseudomonas aeruginosa*, were inexpensive, and had no harmful side effects [19, 23]. The current investigation found that an ethanolic extract of the root of *C. humilis* had potent growth inhibitory properties against multiresistant Gram-positive and Gram-negative bacteria. A very recent study investigated the antimicrobial activity of hydroethanolic extracts from *C. humilis* roots and leaves against five pathogenic microbial strains (2 *E. coli*, 1 *coagulase-negative Staphylococcus*, *Klebsiella pneumoniae*, and *Candida albicans*) using two techniques: microdilution and agar disk diffusion. The results showed that the extracts had an effect against all tested strains, with inhibitory zone values ranging from 12 to 15 mm. The root extract showed the strongest activity against coagulase-negative *Staphylococcus* (IC_50_ = 6.25 mg/mL) [21]. Other findings given by Al-Rimawi et al. [29] looked at the antibacterial activity of methanol and ethanol extracts from *P. halepensis* Mill. Seeds, bark, and cones against *S. aureus* and *E. coli*. Although a pomegranate antimicrobial study reveals the antibacterial process, the active ingredient inside the pomegranate causes toxicity in bacteria by changing and interacting with enzymes and substrates [30]. In contrast, *C. humilis* has been shown to be rich in sesquiterpene lactones [31], which are known for their high antibacterial potential [32].

The use of silver sulfadiazine as a control positive (SDA group) in this study did not produce the best results in terms of collagen creation; instead, it produced the worst results. The unfavorable effects of silver sulfadiazine that are clearly evident in this experiment include wound contracture retardation and delayed and incomplete epithelialization, which are also reported in a work by Muller et al. [33]. The risk of delayed wound healing is increased when the inflammatory process is prolonged and severe. Skin layer renewal is one of the most significant indicators of burn wound healing during therapy [34]. According to our histological analysis, *C. humilis* extract therapy showed regenerative effects on the dermal layers. Recently, several studies have reported the discovery of natural compounds, including those derived from medicinal plants, for the creation of new burn wound healing drugs. In this context, our study attempted to assess the burn wound healing of an ointment made from a Moroccan medicinal plant that is well known for treating skin burns. Natural herbal materials are used in Asian and Indian medicine because they have advantages over manufactured medications. The inclusion of numerous active ingredients in herbal medications may potentially broaden the therapeutic range and improve treatment outcomes [35]. However, no research on the efficacy of *C. humilis* ointment against burn wounds has been performed, according to the literature. Our research looked at the healing of burn wounds in this example. The impact of *C. humilis* extract on rats developing burn wounds was remarkable.

In the research conducted by Akhoondinasab et al. [36], the aloe vera group had a considerably shorter healing time in grade 2 wounds than the silver sulfadiazine group. Because contraction plays a large role in second-degree wounds, especially in rats' slack skin, this impact was more noticeable in the second-degree burn. In our situation, the contraction rate was greater in the EECH and EACH groups than the SDA and control groups. Furthermore, epithelialization is a critical component of wound healing and determines its success. Reepithelialization is required for a wound to be deemed healed. Histological data showed that all of the treated groups had a higher rate of epithelialization than the control group.

Inflammation has three functions: clearing damaged cells, vasodilation, and inflammatory cell extravasation. In our investigation, *C. humilis* ointment had a significant effect on collagen and inflammatory cells. It was comparable to earlier findings, which showed that applying avocado and jojoba oil topically to rats increased collagen production and reduced the number of inflammatory cells during wound healing [37–39].

Inflammation can also aid in the production and degeneration of dermal cells. According to these facts, burn wounds, which are subject to a high level of inflammation and cannot be avoided, should be treated with anti-inflammatory and antioxidant drugs to reduce the risk of delayed wound healing [7]. As proven in our previous work [13], *C. humilis* has a variety of active compounds, each of which can operate as an antibacterial, anti-inflammatory, and powerful antioxidant.

The deposition of collagen in the burn skin was higher in the EECH-treated group's wounds. Indeed, epithelialization was more prominent in the wounds treated with *C. humilis* extracts. Other honey treatment results from Sushma et al. [40] revealed that all treated groups had better collagen deposition at the wound site than the control group. The collagen organization in the treated groups was ordered and horizontally interwoven. In contrast, the collagen fibers in the control group were arranged obliquely and loosely. Angiogenesis is one of the most researched processes in a burnt wound [41].

The results of excellent elastic fibers and minimal angiogenesis production were associated with an effective burn wound healing process in this study. Collagen production is significantly boosted when the dosage of *C. humilis* extract is raised to 20% ointments. In comparison to control and standardization of silver sulfadiazine, this result shows that ointment generated from *C. humilis* extract with 20% ointments may establish an excellent process of burn wound healing in rats. According to these findings, using a 20 percent ointment of EACH and EECH extract to assist an ideal burn wound healing process likely provides a synergic mechanism.

It is crucial to highlight that the extracts did not cause discomfort or pain to the rats throughout the brief duration of wound therapy, since the rats showed no symptoms of restlessness or scratching or biting of the wound site when the extracts were administered.

## 5. Conclusion

The antibacterial activity of crude extracts of *C. humilis* as well as wound healing capabilities was verified in this investigation. In the animals with acute dermal toxicity, there are no treatment-related signs of toxicity or death. The extracts of these plants, on the other hand, might be turned into phytomedicines for the treatment of infected wounds. The results demonstrated that EACH and EECH ointments are successful in treating second-degree burns in rats and may be suitable for short-term therapeutic treatment of second-degree burns. However, other investigations should be conducted to determine the chemical compounds of plant extracts as well as to investigate their antibacterial and wound healing properties. Moreover, other toxicological studies should be investigated to confirm the safety of plant extracts and their bioactive compounds.

## Figures and Tables

**Table 1:** Ingredients for 70 g of ointment for each plant.

Raw materials	Quantity (g)
Plant extract	14
Purified water	10
Lanolin	10
Vaseline	36

**Table 2:** Evaluation of the antimicrobial activity of *C. humilis*.

MIC (mg/mL)	*Staphylococcus aureus*CIP 483	*Bacillus subtilis*CIP 5262	*Escherichia coli*CIP 53126	*Salmonella enterica*CIP 8039	*Pseudomonas aeroginosa*CIP 82118
EECH	2	2	2	2	2
EACH	4	4	4	4	4
Chloramphenicol (25 mg/mL)	0.05	0.05	0.095	0.05	0.095

## Data Availability

The data used to support the findings of this study are included within the article.

## Additional Points

The authors declare that they have no patient data used in this study and declare they have followed the protocols of their work center on the publication of data.
